# Genetic variants in *CYP11B1* influence the susceptibility to coronary heart disease

**DOI:** 10.1186/s12920-022-01307-8

**Published:** 2022-07-13

**Authors:** Xiaoli Huang, Yimin Cheng, Na Wang

**Affiliations:** 1 The Department of Cardiovascology, Xi’an Hospital of Traditional Chinese Medicine, No. 69, Fengcheng Eighth Road, Weiyang District, Xi’an, 710021 People’s Republic of China; 2 The Department of Obstetrics and Gynecology, The Hospital of Xi’an Shiyou University, Xi’an, 710065 People’s Republic of China

**Keywords:** Coronary heart disease, *CYP11B1*, Polymorphisms, Susceptibility, Gene

## Abstract

**Background:**

Genetic factors are important risk factors to develop coronary heart disease (CHD). In this study, we mainly explored whether *CYP11B1* mutations influence CHD risk among Chinese Han population.

**Methods:**

Six variants were genotyped using Agena MassARRAY system from 509 CHD patients and 509 healthy controls. The correlations between *CYP11B1* mutations and CHD risk were assessed using odds ratio (OR) and 95% confidence interval (95% CI) by logistic regression. The haplotype analysis and were ultifactor dimensionality reduction (MDR) were conducted.

**Results:**

In the overall analysis, *CYP11B1* polymorphisms were not correlated with CHD susceptibility. In the stratified analysis, we found that rs5283, rs6410, and rs4534 are significantly associated with susceptibility to CHD dependent on age and gender (*p* < 0.05). Moreover, we also observed that rs5283 and rs4534 could affect diabetes/hypertension risk among CHD patients (*p* < 0.05). In addition, the C_rs4736312_A_rs5017238_C_rs5301_G_rs5283_T_rs6410_C_rs4534_ haplotype of *CYP11B1* reduce the susceptibility to CHD (*p* < 0.05).

**Conclusions:**

We found that rs4534, rs6410 and rs5283 in *CYP11B1* gene influence the susceptibility to CHD, which depend on age and gender.

**Supplementary Information:**

The online version contains supplementary material available at 10.1186/s12920-022-01307-8.

## Introduction

Coronary heart disease (CHD) is a heart disease caused by coronary artery atherosclerosis that causes stenosis or occlusion of the lumen, leading to myocardial ischemia, hypoxia, or necrosis. Epidemiological research showed that more than 8.14 million people died of CHD in 2013, accounting for 50% of the total deaths from cardiovascular disease (CVD) [1]. CHD is considered to be one of the leading causes of death in people worldwide. The World health organization (WHO) predicts that CHD will account for 13.1% of all deaths by 2030 [2]. In China, CHD is the second leading cause of death from CVD, accounting for 22% of urban and 13% of rural mortality. Moreover, the medical costs for CHD are expected to increase by 100%, posing a severe socio-economic burden on individuals and society [3]. Therefore, it is urgent to explore the pathogenesis and etiology of CHD.

It is widely known that CHD is a complex disease that is influenced by environmental and genetic factors [4]. Genetic factors are important risk factors to develop CHD, accounting for as 30 ~ 60% of the variation in the risk of CHD [5]. Large-scale studies have documented that genetic polymorphisms are significantly associated with CHD at genome-wide significance [6]. Moreover, previous studies also have reported that a significant association between candidate gene polymorphism and CHD susceptibility [7–9]. These results underscored the crucial role of genetic factors in the pathogenesis of CHD.

The 11β-hydroxylase (*CYP11B1*) gene is located on chromosome 8q24.3, contains 9 exons and 8 introns, and consists of 503 amino acids [10]. It is a key enzyme responsible for the final step in cortisol biosynthesis [11]. At present, studies have found that the genetic variation of *CYP11B1* was involved in the occurrence and development of various diseases. Rs6410 was significantly related to the secretion of aldosterone in the Chinese population [11]. However, rs4534, rs5283, rs4736312, rs5017238 and rs5301 in *CYP11B1* have not been reported to be related to disease in the literature. The *CYP11B* family is involved in the synthesis of important steroid hormones. The genotypes of the *CYP11B1*/*CYP11B2* loci are in strong linkage disequilibrium [12]. The two synthesize 11β hydroxylase and aldosterone synthase, and then synthesize steroids, which play an important role in myocardial fibrosis, hypertension and arteriosclerosis [10, 13]. Studies have shown that steroid metabolism induces the development of coronary artery disease [14]. *CYP11B1* activates the pituitary-adrenal axis by synthesizing 11β hydroxylase, promoting the accumulation of adrenal cortical hormones, and affecting the production of aldosterone [11], which can damage cardiac or renal organs and induce hypertension [15]. Aldosterone synthase inhibitors can efficiently regulate aldosterone levels [16] by targeting *CYP11B1* with high selectivity. In addition, hypertension and diabetes are major risk factors for cardiovascular disease [15]. Based on the above studies, we hypothesized that *CYP11B1* may be play a vital role in the occurrence of CHD.

Therefore, we explored the associations of *CYP11B1* polymorphisms with CHD susceptibility. These genetic variants may provide a new screening strategy for CHD among Chinese Han population.

## Materials and methods

### Study population

This study recruited 509 patients with CHD and 509 healthy controls from Xi’an Hospital of Traditional Chinese Medicine. The patients were diagnosed as CHD by two experienced imaging specialists according to coronary angiography examination. CHD was defined as more than 1 ( ≥) atherosclerotic plaque in a major coronary artery (≥ 1.5 mm lumen diameter) causing ≥ 50% luminal diameter stenosis by coronary angiography examination. Patients suffered from congenital heart disease, cardiomyopathy, malignancy, chronic inflammatory disease, and liver or kidney disease were excluded. The healthy controls were selected from the same hospital during the same period. The subjects without cardiovascular disease, autoimmune disease, malignancy, and other known disease were included in this study. The study got approval of the ethics committee of the hospital and the experimental procedures were in accordance with the Declaration of Helsinki. Meanwhile, the informed consent signed by the subjects was obtained. Dyslipidemia is a key factor leading to the development of coronary heart disease, and coronary heart disease is related to various indicators such as triglycerides (TG), total cholesterol (TC), low density lipoprotein (LDL) and high density lipoprotein (HDL) in blood lipids [17]. There is an association between elevated serum UA levels and cardiovascular diseases such as coronary heart disease and stroke [18]. In addition, WBC often serves as an effective marker of inflammation, and elevated WBC counts are associated with common risk factors for coronary heart disease, including hypertension. An automatic blood analyzer was used to detect TC, TG, LDL, HDL and other indicators at 4 degrees Celsius. EDTA-treated anticoagulated samples are used to measure blood rheology parameters such as red blood cells (RBC) and white blood cells (WBC).

### SNP selection and genotyping

According to the criteria of minor allele frequency (MAF) ≥ 0.05, six SNPs (rs4534, rs5283, rs6410, rs4736312, rs5017238 and rs5301) of *CYP11B1* gene were selected from dbSNP database (https://www.ncbi.nlm.nih.gov/SNP/). Genomic DNA from peripheral blood was isolated using the DNA Extraction Kit (GoldMag, Co, Ltd, Xi’an, China). The concentration and purity of DNA were measured by NanoDrop 2000 (Thermo Scientific, USA). The selected SNPs were genotyped using Agena MassARRAY system (Agena, San Diego, CA, U.S.A.) as described previously [19, 20] and data was managed using Agena Typer 4.0 software.

### Statistical analysis

Student t-test and χ^2^ test were used to assess the difference in age and gender of study population. The Hardy–Weinberg equilibrium (HWE) in controls was evaluated by χ^2^ test. The associations between *CYP11B1* SNPs and CHD susceptibility was analyzed using odds ratio (OR) and 95% confidence interval (CI) by logistic regression. In addition, linkage disequilibrium (LD) and haplotype analysis were evaluated by the Haploview software and the PLINK software. Multifactor dimensionality reduction (MDR) was conducted to assess the SNP-SNP interactions in the risk of CHD. The differences in clinical parameters in CHD patients were tested by one-way analysis of variance (ANOVA). Statistical power and false positive report probability (FPRP) values were calculated by the Excel spreadsheet which was offered on Wacholder’s website [21]. A *p* < 0.05 was used as the threshold of significance.

## Results

### General characteristics of CHD patients and healthy controls

A total of 509 CHD patients (335 males and 174 females) and 509 healthy controls (335 males and 174 females) included in this study were of Han Chinese ethnicity. The basic characteristics of participants were summarized in Table 1. No significant difference was observed in terms of age (*p* = 0.094), gender (*p* = 1.000), low-density lipoprotein (LDL,* p* = 0.157), urea (*p* = 0.056) between the case and control groups, with their mean age being 62.16 ± 10.30 and 61.14 ± 9.02 years, respectively. Among all the CHD patients, 318 patients had hypertension, and 147 patients had diabetes. Compared with the normal control group, the levels of TG, TC, HDL, UA, RBC, WBC, HGB and Platelet were significantly decreased in the CHD group.

**Table 1:** Primary characteristics of the cases and controls

Variants	Case (N = 509)	Control (N = 509)	*p* value
Age, years	62.16 ± 10.30	61.14 ± 9.02	0.094^a^
> 60 (N, %)(N, %)	283 (55.6%)	284 (55.8%)	
≤ 60 (N, %)	226 (44.4%)	225 (44.2%)	
Gender			1.000^b^
Male (N, %)	335 (65.8%)	335 (65.8%)	
Female (N, %)	174 (34.2%)	174 (34.2%)	
TG (mmol/L)	1.62 ± 1.00	1.84 ± 1.46	**0.006**^**a**^
TC (mmol/L)	4.08 ± 1.08	4.76 ± 0.91	** < 0.001**^**a**^
LDL (mmol/L)	3.83 ± 1.92	2.60 ± 0.73	0.157 ^a^
HDL (mmol/L)	1.12 ± 0.26	1.17 ± 0.36	**0.016 **^**a**^
UA (μ mol/L)	300.90 ± 92.21	320.41 ± 79.40	**0.001**^**a**^
Urea	5.37 ± 2.14	5.15 ± 1.31	0.056^a^
RBC	4.82 ± 0.46	4.22 ± 0.97	** < 0.001**^**a**^
WBC	10.75 ± 4.52	5.81 ± 1.51	** < 0.001**^**a**^
Platelet (10^9^/L)	183.04 ± 74.38	213.73 ± 59.60	** < 0.001**^**a**^
HGB	129.73 ± 28.98	147.39 ± 16.33	** < 0.001**^**a**^
Hypertension (N, %)	318 (62.5%)		
Diabetes (N, %)	147 (28.9%)		

Numbers in **bold** mean statistical significance

*TG* triglyceride; *TC* total cholesterol; *LDL* low-density lipoprotein; *HDL* high-density lipoprotein; *UA* uric acid; *RBC* red blood cells; *WBC* white blood cells; *HGB* hemoglobin

*p* ^a^ and *p* ^b^ values were calculated by *t*-test and χ^2^ test, respectively

### Association of *CYP11B1* polymorphisms and CHD risk

Additional File 1: Table S1 exhibited the primary information of *CYP11B1* polymorphisms. The genotype frequency distributions of the selected polymorphisms of *CYP11B1* were in line with HWE (*p* > 0.05).

We investigated the association between *CYP11B1* polymorphisms and CHD risk among Chinese Hans. As shown in Table 2, *CYP11B1* polymorphisms were not correlated with CHD susceptibility in total population under five heritance models (*p* > 0.05).

**Table 2:** Association of *CYP11B1* polymorphisms and CHD risk

SNP	Model	Genotype	Case (N, %)	Control (N, %)	OR (95% CI)	*p* value^a^
rs4534	Allele	C	592 (58.50%)	610 (60.04%)	1.00	
		T	420 (41.50%)	406 (39.96%)	1.07(0.89–1.27)	0.480
	Codominant	CC	165 (32.61%)	181 (35.63%)	1.00	
	Homozygote	TT	79 (15.61%)	79 (15.55%)	1.11 (0.76 -1.62)	0.582
	Heterozygote	TC	262 (51.78%)	248 (48.82%)	1.16(0.88–1.53)	0.279
	Dominant	CC	165 (32.61%)	181 (35.63%)	1.00	
		TT + TC	341 (67.39%)	327 (64.37%)	1.15 (0.89 -1.49)	0.290
	Recessive	TC + CC	427 (84.39%)	429 (84.45%)	1.00	
		TT	79 (15.61%)	79 (15.55%)	1.02(0.72–1.43)	0.928
	Additive	–	–	–	1.08(0.90 -1.29)	0.432
rs5283	Allele	G	703 (69.33%)	717 (70.715)	1.00	
		A	311 (30.67%)	297 (29.29%)	1.07(0.88–1.29)	0.497
	Codominant	GG	240 (47.33%)	257 (50.69%)	1.00	
	Homozygote	AA	44 (8.68%)	47 (9.27%)	0.99 (0.63–1.56)	0.978
	Heterozygote	AG	223 (43.99)	203 (40.03)	1.18 (0.91–1.53)	0.214
	Dominant	GG	240 (47.33%)	257 (50.69%)	1.00	
		AA + AG	267 (52.67%)	250 (49.31%)	1.14(0.89–1.46)	0.286
	Recessive	AG + GG	463 (91.32%)	460 (90.73%)	1.00	
		AA	44 (8.68%)	47 (9.27%)	0.92(0.60–1.42)	0.708
	Additive	–	–	–	1.07 (0.88–1.29)	0.510
rs6410	Allele	C	741 (73.08%)	712 (70.22%)	1.00	
		T	273 (26.92%)	306 (30.18%)	0.86(0.71–1.04)	0.117
	Codominant	CC	271 (53.45%)	245 (48.13%)	1.00	
	Homozygote	TT	37 (7.30%)	42 (8.25%)	0.79 (0.49 -1.26)	0.320
	Heterozygote	TC	199 (39.25%)	222 (43.62%)	0.80(0.62–1.04)	0.100
	Dominant	CC	271 (53.45%)	245 (48.13%)	1.00	
		TT + TC	236 (46.55%)	264 (58.17%)	0.80(0.63–1.03)	0.080
	Recessive	TC + CC	470 (92.70%)	467 (91.75%)	1.00	
		TT	37 (7.30%)	42 (8.25%)	0.87(0.55–1.37)	0.540
	Additive	–	–	–	0.85 (0.70 -1.03)	0.101
rs4736312	Allele	C	850 (83.83%)	853 (83.96%)	1.00	
		A	164 (16.17%)	163 (16.04%)	1.01(0.80–1.28)	0.936
	Codominant	AA	356 (70.22%)	357 (20.28%)	1.00	
	Homozygote	CC	13 (2.56%)	12 (2.36%)	1.06(0.48–2.36)	0.888
	Heterozygote	CA	138 (27.22%)	139 (27.36%)	0.99(0.75–1.30)	0.926
	Dominant	AA	356 (70.22%)	357 (70.28%)	1.00	
		CC + CA	151 (29.78%)	151 (29.72%)	0.99(0.76–1.30)	0.957
	Recessive	CA + AA	494 (97.44%)	496 (97.64%)	1.00	
		CC	13 (2.56%)	12 (2.36%)	1.06(0.48–2.36)	0.880
	Additive	–	–	–	1.00(0.79–1.27)	0.998
rs5017238	Allele	A	838 (83.47%)	850 (83.83%)	1.00	
		G	166 (16.53%)	164 (16.17%)	1.03(0.81–1.30)	0.827
	Codominant	AA	356 (70.92%)	357 (70.41%)	1.00	
	Homozygote	GG	20 (3.98%)	14 (2.76%)	1.40(0.69–2.82)	0.349
	Heterozygote	GA	126(25.10%)	136 (26.82%)	0.92(0.69–1.22)	0.573
	Dominant	AA	356 (70.92%)	357 (70.41%)	1.00	
		GG + GA	146 (29.08%)	150 (29.59%)	0.97(0.74–1.27)	0.804
	Recessive	GA + AA	482 (96.02%)	493 (97.24%)	1.00	
		GG	20 (3.98%)	14 (2.76%)	1.43(0.71–2.87)	0.315
	Additive	–	–	–	1.02(0.81–1.28)	0.898
rs5301	Allele	C	841 (83.10%)	853 (83.79%)	1.00	
		T	171 (16.90%)	165 (16.21%)	1.05(0.83–1.33)	0.676
	Codominant	CC	349 (68.97%)	356 (69.94%)	1.00	
	Homozygote	TT	14 (2.77%)	12 (2.36%)	1.16(0.53–2.55)	0.709
	Heterozygote	TC	143 (28.26%)	141 (27.70%)	1.03(0.78–1.36)	0.845
	Dominant	CC	349 (68.97%)	356 (69.94%)	1.00	
		TT + TC	157 (31.03%)	153 (30.06%)	1.04(0.79–1.36)	0.783
	Recessive	TC + CC	492 (97.23%)	497 (97.64%)	1.00	
		TT	14 (2.77%)	12 (2.36%)	1.15(0.53–2.52)	0.723
	Additive	–			1.04(0.82–1.32)	0.726

*SNP* single nucleotide polymorphism; *OR* odds ratio; *CI* confidence interval

*p* < 0.05 indicates statistical significance

^*a*^Adjusted for age and gender

### Stratification analyses

The correlation between *CYP11B1* polymorphisms and CHD risk was further analyzed in different subgroups (age, gender, hypertension and diabetes). Table 3 showed that the TC (OR = 1.80, 95% CI = 1.19–2.74, *p* = 0.006) and TT + TC genotypes (OR = 1.68, 95% CI = 1.13–2.50, *p* = 0.011) of rs4534 was related to a significantly increased risk of CHD in younger population (age ≤ 60 years). However, the T allele (OR = 0.72, 95% CI = 0.53–0.96, *p* = 0.026), TC genotype (OR = 0.67, 95% CI = 0.45–0.99, *p* = 0.042), TT + TC genotype (OR = 0.65, 95% CI = 0.45–0.94, *p* = 0.023) and the additive model (OR = 0.70, 95% CI = 0.52–0.95, *p* = 0.022) of rs6410 showed a decreased risk of CHD patients with age ≤ 60 years.

**Table 3:** The association between *CYP11B1* polymorphisms and CHD susceptibility stratified by age and gender

Age										
SNP	Model	Genotype	> 60 years				≤ 60 years			
			Case (N, %)	Control (N, %)	OR(95% CI)	*p*	Case (N, %)	Control (N, %)	OR(95% CI)	*p*
rs6410	Allele	C	401 (71.10%)	402 (70.77%)	1.00		340(75.56%)	310 (68.89%)	1.00	
		T	163 (28.90%)	166 (29.23%)	0.98(0.76–1.27)	0.904	110 (24.44%)	140 (31.11%)	0.72(0.53–0.96)	**0.026**
	Codominant	CC	144 (51.06%)	142 (50.00%)	1.00		127 (56.45%)	103 (45.78%)	1.00	
		TT	25 (8.87%)	24 (8.45%)	1.01(0.55–1.87)	0.970	12 (5.33%)	18 (8.00%)	0.54(0.25–1.18)	0.122
		TC	113 (40.07%)	118 (41.55%)	0.89(0.63–1.27)	0.525	86 (38.22%)	104 (46.22%)	0.67(0.45–0.99)	0.042
	Dominant	CC	144 (51.06%)	142 (50.00%)	1.00		127 (56.45%)	103 (45.78%)	1.00	
		TT + TC	138(48.94%)	130 (50.00%)	0.91(0.65–1.27)	0.589	98(43.55%)	122 (54.22%)	0.65(0.45–0.94)	**0.023**
	Recessive	TC + CC	257 (91.13%)	260 (91.55%)	1.00		213 (94.67%)	207 (92.00%)	1.00	
		TT	25 (8.87%)	24 (8.45%)	1.07(0.59–1.93)	0.836	12 (5.33%)	18 (8.00%)	0.65(0.30–1.38)	0.258
	Additive	–	–	–	0.96(0.74–1.24)	0.743	/	/	0.70(0.52–0.95)	**0.022**
rs4534	Allele	C	341 (60.68%)	337 (59.33%)	1.00		251 (55.78%)	273 (60.94%)	1.00	
		T	221 (39.32%)	231 (40.67%)	0.95(0.75–1.20)	0.644	199 (44.22%)	175 (39.06%)	1.24(0.95–1.61)	0.117
	Codominant	CC	104 (37.01%)	95 (33.45%)	1.00		61 (27.11%)	86 (38.39%)	1.00	
		TT	44 (15.66%)	42 (14.79%)	0.99(0.59–1.65)	0.956	35 (15.56%)	37 (16.52%)	1.33(0.76–2.35)	0.321
		TC	133 (47.33%)	147 (51.76%)	0.82(0.56–1.18)	0.279	129 (57.33%)	101 (45.09%)	1.80(1.19–2.74)	**0.006**
	Dominant	CC	104 (37.01%)	95 (33.45%)	1.00		61 (27.11%)	86 (38.39%)	1.00	
		TT + TC	177 (62.99%)	189 (66.55%)	0.85(0.60–1.21)	0.372	164 (72.89%)	138 (61.61%)	1.68(1.13–2.50)	**0.011**
	Recessive	TC + CC	237 (84.34%)	233 (85.21%)	1.00		190 (84.44%)	187 (83.48%)	1.00	
		TT	44 (15.66%)	42 (14.79%)	1.11(0.70–1.77)	0.658	35 (15.56%)	37 (16.52%)	0.93(0.56–1.54)	0.780
	Additive	–	–	–	0.95(0.74–1.22)	0.694	/	/	1.25(0.95–1.65)	0.108

Gender										
Gene SNP	Model	Genotype	Male				Female			
			Case (N, %)	Control (N, %)	OR(95% CI)	*p*	Case (N, %)	Control (N, %)	OR(95% CI)	*p*
rs5283	Allele	G	464 (69.46%)	458 (68.77%)	1.00		374 (68.00%)	259 (74.43%)	1.00	
		A	204 (30.54%)	208 (31.23%)	0.97(0.77–1.22)	0.784	176 (32.00%)	89 (25.57%)	1.37(1.01–1.85)	**0.040**
	Codominant	GG	163 (48.80%)	157 (47.15%)	1.00		118 (42.91%)	100 (57.47%)	1.00	
	Homozygote	AA	33 (9.88%)	32 (9.61%)	0.99(0.58–1.69)	0.977	19 (6.91%)	15 (8.62%)	1.08(0.52–2.24)	0.838
	Heterozygote	AG	138 (41.32%)	144 (43.24%)	0.92(0.67–1.27)	0.629	138 (50.18%)	59 (33.91%)	1.98(1.32–2.97)	**0.001**
	Dominant	GG	163 (48.80%)	157 (47.15%)	1.00		118 (42.91%)	100 (57.47%)	1.00	
		AA + AG	171(51.20%)	176 (52.85%)	0.94(0.69–1.27)	0.672	157 (57.09%)	74 (42.53%)	1.80(1.23–2.64)	**0.003**
	Recessive	AG + GG	301 (90.12%)	301 (90.39%)	1.00		256 (93.09%)	159 (91.38%)	1.00	
		AA	33 (9.88%)	32 (9.61%)	1.03(0.62–1.72)	0.912	19 (6.91%)	15 (8.62%)	0.79(0.39–1.60)	0.514
	Additive	–	–	–	0.97(0.77–1.22)	0.786	/	/	1.40(1.03–1.91)	**0.034**
rs6410	Allele	C	478 (71.56%)	478 (71.34%)	1.00		413 (75.09%)	234 (67.24%)	1.00	
		T	190 (28.44%)	192 (28.66%)	0.99(0.78–1.26)	0.931	137 (24.91%)	114 (32.76%)	0.68(0.51–0.92)	**0.011**
	Codominant	CC	176 (52.70%)	169 (50.44%)	1.00		152 (55.27%)	76 (43.68%)	1.00	
	H omozygote	TT	32 (9.58%)	26 (7.76%)	1.17(0.67–2.06)	0.573	14 (5.09%)	16 (9.20%)	0.44(0.20–0.95)	**0.036**
	Heterozygote	TC	126 (37.72%)	140 (41.20%)	0.86(0.62–1.19)	0.357	109 (39.64%)	82 (47.12%)	0.67(0.45–0.99)	**0.044**
	Dominant	CC	176 (52.70%)	169 (50.44%)	1.00		152 (55.27%)	76 (43.68%)	1.00	
		TT + TC	158 (47.30%)	166 (49.56%)	0.91(0.67–1.23)	0.539	123 (44.73%)	98 (56.32%)	0.63(0.43–0.92)	**0.017**
	Recessive	TC + CC	302 (90.42%)	309 (92.24%)	1.00		261 (94.91%)	158 (90.80%)	1.00	
		TT	32 (9.58%)	26 (7.76%)	1.25(0.73–2.16)	0.412	14 (5.09%)	16 (9.20%)	0.53(0.25–1.12)	0.096
	Additive	–	–	–	0.99(0.78–1.25)	0.907	/	/	0.66(0.49–0.90)	**0.009**

*SNP* single nucleotide polymorphism; *OR* odds ratio; *95% CI* 95% confidence interval

*p* values were calculated by logistic regression analysis with adjustment for age and gender

**Bold** values indicate statistical significance (*p* < 0.05)

The results of gender stratification were presented in Table 3. In females, except for the recessive model, rs6410 was correlated with a lower-risk of CHD in other models (allele: OR = 0.68, 95% CI = 0.51–0.92, *p* = 0.011; homozygote: OR = 0.44, 95% CI = 0.20–0.95, *p* = 0.036; heterozygote: OR = 0.67, 95% CI = 0.45–0.99, *p* = 0.044; dominant: OR = 0.63, 95% CI = 0.43–0.92, *p* = 0.017; additive: OR = 0.66, 95% CI = 0.49–0.90, *p* = 0.009). However, rs4583 could elevate the susceptibility to CHD in the allele (OR = 1.37, 95% CI = 1.01–1.85, *p* = 0.040), heterozygote (OR = 1.98, 95% CI = 1.32–2.97, *p* = 0.001), dominant (OR = 1.80, 95% CI = 1.23–2.64, *p* = 0.003), and additive model (OR = 1.40, 95% CI = 1.03–1.91, *p* = 0.034).

Stratified analyses by diabetes revealed that rs5283 increased the risk of diabetes in CHD patients (allele: OR = 1.45, 95% CI = 1.09–1.93, *p* = 0.012; heterozygote: OR = 1.79, 95% CI = 1.19–2.70, *p* = 0.006; dominant: OR = 1.78, 95% CI = 1.20–2.64, *p* = 0.004; additive: OR = 1.47, 95% CI = 1.09–1.98, *p* = 0.011, Table 4). While rs4534 could reduce the risk of diabetes in CHD subjects (allele: OR = 0.74, 95% CI = 0.56–0.98, *p* = 0.032; homozygote: OR = 0.53, 95% CI = 0.28–0.99, *p* = 0.048; additive: OR = 0.73, 95% CI = 0.54–0.98, *p* = 0.036). In addition, we found rs4534 was associated with a higher risk of hypertension in CHD patients (homozygote: OR = 1.97, 95% CI = 1.08–3.59, *p* = 0.026; recessive: OR = 1.84, 95% CI = 1.07–3.16, *p* = 0.028; additive: OR = 1.33, 95% CI = 1.01–1.75, *p* = 0.044). There were no significant associations between rs4736312, rs5017238, and rs5301 and CHD susceptibility.

**Table 4:** Associations of *CYP11B1* polymorphisms and CHD risk stratified by diabetes and hypertension

SNP	Model	Genotype	Diabetes				Hypertension			
			Case (N, %)	Control (N, %)	OR(95% CI)	*p*	Case (N, %)	Control (N, %)	OR(95% CI)	*p*
rs5283	Allele	G	187 (63.61%)	516 (71.67%)	1.00		439 (69.24%)	264 (69.47%)	1.00	
		A	107 (36.39%)	204(28.33%)	1.45(1.09–1.93)	**0.012**	195 (30.76%)	116 (30.53%)	1.01(0.77–1.33)	0.939
	Codominant	GG	55 (37.42%)	185 (51.39%)	1.00		150 (47.32%)	90 (47.37%)	1.00	
		AA	15 (10.20%)	29 (8.06%)	1.73(0.86–3.46)	0.123	28 (8.83%)	16 (8.42%)	1.06(0.54–2.08)	0.868
		AG	77 (52.38%)	146 (40.55%)	1.79(1.19–2.70)	**0.006**	139 (43.85%)	84 (44.21%)	0.98(0.67–1.44)	0.921
	Dominant	GG	55 (37.42%)	185 (51.39%)	1.00		150 (47.32%)	90 (47.37%)	1.00	
		AA + AG	92 (62.58%)	175 (48.61%)	1.78(1.20–2.64)	**0.004**	167 (52.68%)	100 (52.63%)	0.99(0.69–1.4)	0.972
	Recessive	AG + GG	132 (89.08%)	331 (91.94%)	1.00		289 (91.17%)	174 (91.58%)	1.00	
		AA	15 (10.20%)	29 (8.06%)	1.29(0.67–2.49)	0.450	28 (8.83%)	16 (8.42%)	1.07(0.56–2.05)	0.842
	Additive	–	–	–	1.47(1.09–1.98)	**0.011**	/	/	1.01(0.76–1.34)	0.952
rs4534	Allele	C	186 (63.70%)	406 (56.39%)	1.00		356 (56.33%)	236 (62.11%)	1.00	
		T	106 (36.30%)	314 (43.61%)	0.74(0.56–0.98)	**0.032**	276 (43.67%)	144 (37.89%)	1.27(0.98–1.65)	0.071
	Codominant	CC	57 (39.04%)	108 (30.00%)	1.00		98 (31.01%)	67 (35.26%)	1.00	
	Homozygote	TT	17 (11.64%)	62 (17.22%)	0.53(0.28–0.99)	**0.048**	58 (18.35%)	21 (11.05%)	1.97(1.08–3.59)	**0.026**
	Heterozygote	TC	72 (49.32%)	190 (52.78%)	0.74(0.48–1.13)	0.157	160 (50.63%)	102 (53.69%)	1.12(0.75–1.68)	0.581
	Dominant	CC	57 (39.04%)	108 (30.00%)	1.00		98 (31.01%)	67 (35.26%)	1.00	
		TT + TC	89 (60.96%)	252 (70.00%)	0.69(0.46–1.03)	0.068	218 (68.99%)	123 (64.74%)	1.26(0.86–1.87)	0.238
	Recessive	TC + CC	129 (88.36%)	298 (82.78%)	1.00		258 (81.65%)	169 (88.95%)	1.00	
		TT	17 (11.64%)	62 (17.22%)	0.64(0.36–1.14)	0.126	58 (18.35%)	21 (11.05%)	1.84(1.07–3.16)	**0.028**
	Additive	–	–	–	0.73(0.54–0.98)	**0.036**	/	/	1.33(1.01–1.75)	**0.044**

*CHD* coronary heart disease; *SNP* single nucleotide polymorphism; *OR* odds ratio; *95% CI* 95% confidence interval

*p* values were calculated by logistic regression analysis with adjustment for age and gender

**Bold** values indicate statistical significance (*p* < 0.05)

### Clinical characteristics and SNPs

We also investigated the correlation between clinical characteristics and SNPs (Additional File 1: Table S2). The results have shown that there was no significant correlation between the genetic variation of *CYP11B1* and clinical parameters (*p* > 0.05).

### FPRP analysis

FPRP and statistical power were calculated for all positive results. As shown in Additional File 1: Table S3, at the prior probability of 0.1 and FPRP threshold of 0.2, the significant results of rs5283 remained noteworthy.

### Haplotype analysis and MDR analysis

**Fig. 1:**
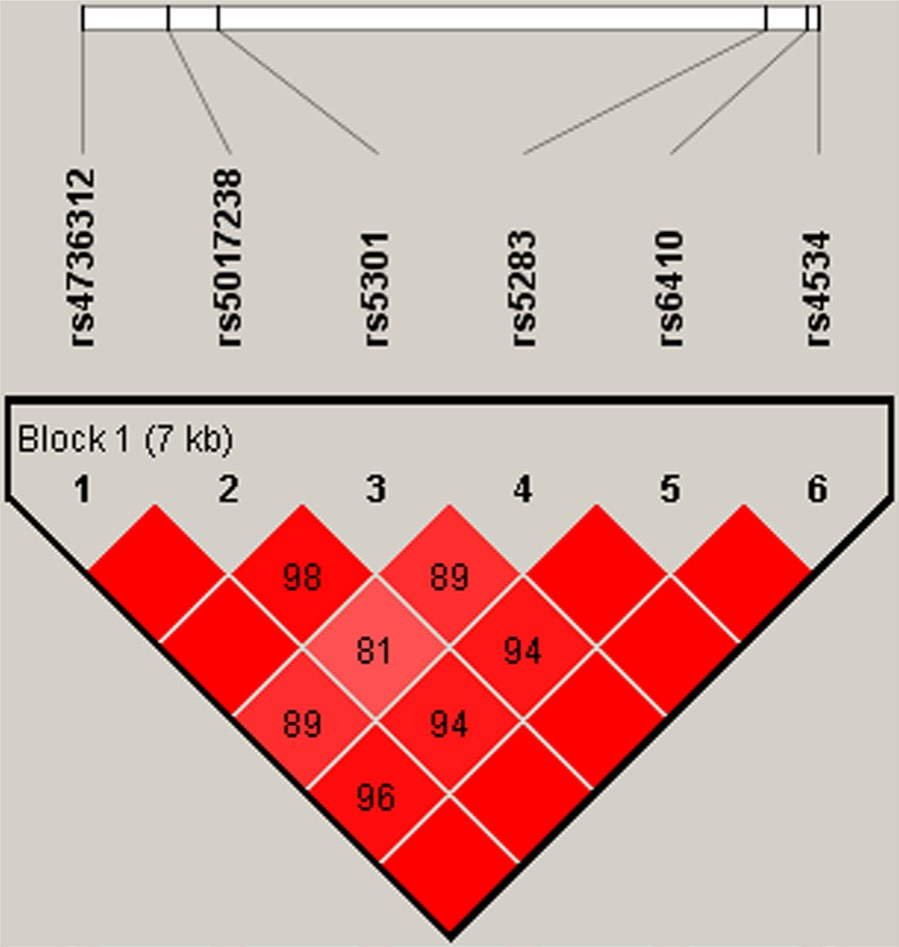
Haplotype block map for SNPs in *CYP11B1. *Block 1 includes rs4736312, rs5017238, rs5301, rs5283, rs6410 and rs4534. The numbers inside the diamonds indicate the D’ for pairwise analyses.

The haplotype analysis of *CYP11B1* polymorphisms and CHD risk was conducted. The results of Table 5 presented that the C_rs4736312_A_rs5017238_C_rs5301_G_rs5283_T_rs6410_C_rs4534_ haplotype was correlated with a decreased risk of CHD compared to C_rs4736312_A_rs5017238_C_rs5301_G_rs5283_C_rs6410_T_rs4534_ (OR = 0.72, 95%CI = 0.54–0.96, *p* = 0.024). And we observed an LD plot consisted of six SNPs (rs4736312, rs5017238, rs5301, rs5283, rs6410 and rs4534), as exhibited in Fig. 1.

**Table 5:** Haplotype analysis of *CYP11B1* polymorphisms and CHD risk

Haplotypes	Frequency in case	Frequency in control	Without adjustment		With adjustment	
			OR(95% CI)	*p* value	OR(95% CI)	*p* value
C_rs4736312_A_rs5017238_C_rs5301_G_rs5283_C_rs6410_T_rs4534_	0.414	0.400	1.00		1.00	
C_rs4736312_A_rs5017238_C_rs5301_A_rs5283_C_rs6410_C_rs4534_	0.304	0.289	1.00 (0.81–1.24)	0.99	1.00 (0.81–1.24)	1.000
A_rs4736312_G_rs5017238_T_rs5301_G_rs5283_T_rs6410_C_rs4534_	0.159	0.157	0.95 (0.74–1.24)	0.73	0.95 (0.73–1.23)	0.690
C_rs4736312_A_rs5017238_C_rs5301_G_rs5283_T_rs6410_C_rs4534_	0.107	0.144	0.72 (0.54–0.96)	**0.027**	0.72 (0.54–0.96)	**0.024**

*CHD* coronary heart disease; *OR* odds ratio; *95% CI*, 95% confidence interval

**Bold** values indicate statistical significance (*p* < 0.05)

**Fig. 2:**
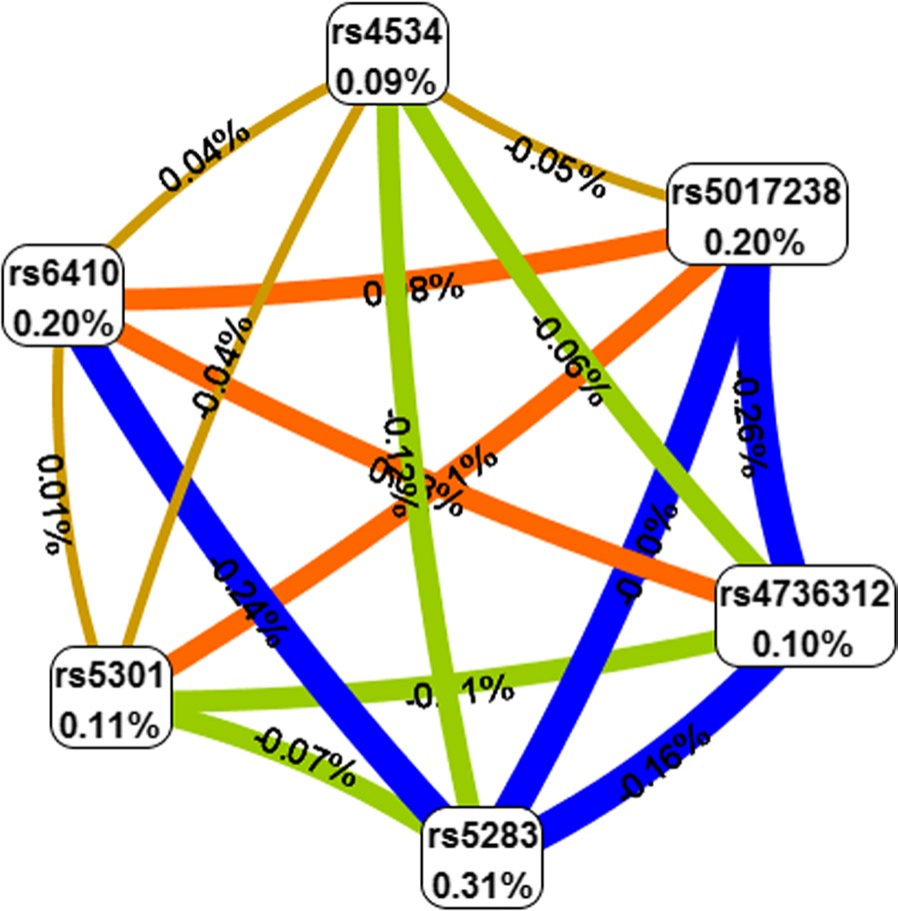
The Fruchterman-Reingold of SNP-SNP interactions. Each SNP is reported in per cent the value of Information Gain (IG), while numbers in the connections indicate the entropy-based IG for the SNP pairs. Orange bar indicate the high-level synergies on the phenotype, while the brown indicate a medium-level interaction, green and blue connections with negative IG values indicate redundancy or lack of synergistic interactions between the markers

Then, the SNP-SNP interaction was performed by MDR analysis. As shown in Fig. 2, the Fruchterman-Reingold (Fig. 2) described the interactions between these SNPs. The results of MDR model analysis of the SNP-SNP interactions are demonstrated in Table 6. The six-locus model including rs4736312, rs5017238, rs5301, rs5283, rs6410, rs4534 was the best model and driving the high-risk combinations for CHD (CVC = 10/10, OR = 1.51, 95% CI = 1.18–1.93, *p* = 0.0011).

**Table 6:** MDR analysis of SNP-SNP interactions

Model	Training Bal. Acc	Testing Bal. Acc	CVC	OR (95% CI)	*p*
rs6410	0.527	0.518	7/10	1.24 (0.97–1.58)	0.091
rs5017238,rs6410	0.542	0.492	7/10	1.39 (1.08–1.79)	**0.009**
rs5017238,rs6410,rs4534	0.550	0.500	7/10	1.46 (1.14–1.87)	**0.003**
rs5017238,rs5301,rs5283,rs4534	0.554	0.503	6/10	1.51 (1.18–1.93)	**0.0011**
rs4736312,rs5017238,rs5301,rs5283,rs4534	0.554	0.503	6/10	1.51 (1.18–1.93)	**0.0011**
rs4736312,rs5017238,rs5301,rs5283,rs6410,rs4534	0.554	0.497	10/10	1.51 (1.18–1.93)	**0.0011**

*MDR* multifactor dimensionality reduction; *Bal. Acc.* balanced accuracy; *CVC* cross–validation consistency; *OR*, odds ratio; *CI*, confidence interval

*p* < 0.05 indicates statistical significance, which was indicated in bold

## Discussion

This is the first study to explore the effect of *CYP11B1* polymorphisms on CHD susceptibility. The results of overall analysis revealed that *CYP11B1* polymorphisms were not correlated with CHD susceptibility. In the stratified analysis, we found that rs5283, rs6410, and rs4534 are significantly associated with susceptibility to CHD in females and individuals aged ≤ 60 years old. Moreover, we also observed that rs5283 and rs4534 could affect diabetes/hypertension risk among CHD patients. In addition, the C_rs4736312_A_rs5017238_C_rs5301_G_rs5283_T_rs6410_C_rs4534_ haplotype of *CYP11B1* reduce the susceptibility to CHD. These data highlight the crucial role of *CYP11B1* genetic variants in the development of CHD.

*CYP11B1* gene is located on chromosome 8q24.3, containing 9 exons and 8 introns. It catalyzes the final step of cortisol biosynthesis. Some studies have documented that elevated cortisol is associated with a number of metabolic changes, such as hyperlipidaemia, diabetes, hypertension and abdominal adiposity [22, 23], which are correlated with CHD risk. Moreover, cortisol had a direct impact on the heart and blood vessels and played an important role in the process of atherogenesis and cardiovascular disease [24]. These results implied that *CYP11B1* may be involved in pathophysiology of CHD through regulating cortisol. Recently, some reports have studied the role of *CYP11B1* polymorphisms in disease. For example, Deng et al. indicated that rs4534 showed no significant association with autism in Chinese children [25]. Zhang et al. have shown that rs6410 was related to primary hyperaldosteronism, which is a common form of secondary hypertension [11]. Meanwhile, Wang et al. indicated that rs6410 and rs6387 haplotype is correlated with persistent postoperative hypertension in Chinese patients undergoing adrenalectomy with aldosterone-producing adenoma [13]. However, no study has investigated the effect of *CYP11B1* genetic variants on CHD. Rs6410 in *CYP11B1* can affect skeletal maturation through variable shearing [26] and was significantly associated with trabecular bone mineral density and cross-sectional area in Caucasian elderly men [27]. However, rs5283, rs4736312, rs5017238 and rs5301 in *CYP11B1* have not been reported to be related to disease in the literature.

In the present study, we found that rs6410 reduced the susceptibility to CHD individuals aged ≤ 60 years old and females. Rs5283 not only increased the susceptibility to CHD in females, but also enhanced the risk of diabetes among CHD patients. Rs4534 also correlated with increased risk of CHD subjects younger than 60 years. Besides, in CHD patients, rs4534 enhanced the susceptibility to diabetes, whereas reduced the risk of hypertension. The rs4534 polymorphism is a missense variant located in exon 9 of the *CYP11B1* gene. Rs5283 and rs6410 are, synonymous nucleotide polymorphisms, located on the exon region. Missense and synonymous mutations have been widely studied in the development of disease by causing changes in protein expression, conformation and function [28–31]. Therefore, we presumed that these three polymorphisms can affect *CYP11B1* gene mRNA and protein by altering translation, mRNA stability or protein folding, thereby affecting CHD susceptibility. However, it should be confirmed in further functional studies.

## Conclusions

To sum up, we found that a missense mutation (rs4534) and two synonymous variants (rs6410 and rs5283) in *CYP11B1* gene influence the susceptibility to CHD, which depend on age and gender. It indicated that *CYP11B1* gene variant plays an important role in the development of CHD. These mutations may provide new ideas for exploring the pathogenesis of CHD.

## Supplementary Information

**Additional file 1:** Basic information on SNPs of *CYP11B1*, clinical characteristics of participants with different SNPs, and false-positive reporting probability of susceptibility.

## Data Availability

The datasets generated and/or analysed during the current study are available in the zenodo repository (https://zenodo.org/record/6562820#.YoXyqPnISUk).

## Abbreviations

CHD: Coronary heart disease
FPRP: False positive report probability
OR: Odds ratio
CI: Confidence intervals
MAF: Minor allele frequency
HWE: Hardy–Weinberg equilibrium
MDR: Multifactor dimensionality reduction
