# Supplementary material for: Genetic variants in CYP11B1 influence the susceptibility to coronary heart disease
Source: BMC Med Genomics. 2022 Jul 13;15:158. doi: 10.1186/s12920-022-01307-8 (PMC9281100; doi:10.1186/s12920-022-01307-8)
Supplement: Supplementary file 1 — Additional file 1: Basic information on SNPs of CYP11B1, clinical characteristics of participants with different SNPs, and false-positive reporting probability of susceptibility. [file 12920_2022_1307_MOESM1_ESM.docx]

Additional File 1: Table S1 Basic information of SNPs of *CYP11B1* in this study

| SNP | Chromosome | Function | Allele (Minor/major) | Protein residue | HWE  *p* |
| --- | --- | --- | --- | --- | --- |
| rs4534 | 8q24.3 | Missense | T/C | Gln,Arg | 0.781 |
| rs5283 | 8q24.3 | Synonymous | A/G | Asp | 0.454 |
| rs6410 | 8q24.3 | Synonymous | T/C | Leu | 0.461 |
| rs4736312 | 8q24.3 | 3’-UTR | A/C | / | 0.869 |
| rs5017238 | 8q24.3 | 3’-UTR | G/A | / | 0.746 |
| rs5301 | 8q24.3 | 3’-UTR | T/C | / | 0.746 |

Abbreviations: SNP, single nucleotide polymorphism; HWE, Hardy-Weinberg equilibrium.

Additional File 1: Table S2 Clinical characteristics of CHD patients based on *CYP11B1* polymorphisms

| Characteristics | **rs5238** | | | | **rs6410** | | | |
| --- | --- | --- | --- | --- | --- | --- | --- | --- |
|  | **AA** | **AG** | **GG** | *p* value | **TT** | **TC** | **CC** | *p* value |
| TG (mmol/L) | 1.76±1.09 | 1.63±1.01 | 1.57±0.97 | 0.498 | 1.54±0.61 | 1.58±1.06 | 1.65±0.99 | 0.678 |
| TC (mmol/L) | 4.24±0.88 | 4.13±1.13 | 4.01±1.06 | 0.325 | 4.01±1.04 | 4.07±1.09 | 4.10±1.08 | 0.865 |
| LDL (mmol/L) | 9.58±45.94 | 2.37±0.87 | 4.11±19.07 | 0.075 | 9.99±44.21 | 2.39±0.92 | 4.02±19.95 | 0.091 |
| HDL (mmol/L) | 1.11±0.22 | 1.13±0.27 | 1.10±0.27 | 0.562 | 1.06±0.27 | 1.13±0.27 | 1.12±0.26 | 0.413 |
| UA (μmol/L) | 316.18±91.20 | 295.56±87.18 | 303.01±96.81 | 0.356 | 317.05±100.37 | 295.93±97.53 | 302.32±86.92 | 0.414 |
| Urea | 5.32±1.64 | 5.36±2.32 | 5.38±2.05 | 0.980 | 5.76±2.38 | 5.27±2.03 | 5.39±2.18 | 0.418 |
| RBC | 4.39±0.91 | 4.20±1.01 | 4.22±0.94 | 0.474 | 4.04±1.10 | 4.20±0.99 | 4.27±0.93 | 0.371 |
| WBC | 11.28±17.27 | 10.98±14.66 | 10.44±13.88 | 0.893 | 13.78±18.24 | 10.08±12.46 | 10.83±15.33 | 0.359 |
| Platelet (10^9^/L) | 182.18±78.07 | 183.69±76.44 | 182.60±72.03 | 0.984 | 166.32±81.70 | 181.20±70.85 | 186.68±75.78 | 0.268 |
| HGB | 132.95±27.77 | 129.58±28.87 | 129.30±29.38 | 0.745 | 126.70±33.83 | 128.35±29.80 | 131.17±27.66 | 0.469 |
| Characteristics | **rs4534** | | | | **rs4736312** | | | |
|  | **TT** | **TC** | **CC** | *p* value | **CC** | **CA** | **AA** | *p* value |
| TG (mmol/L) | 1.53±0.82 | 1.65±1.10 | 1.60±0.91 | 0.626 | 1.55±0.57 | 1.54±0.88 | 1.65±1.05 | 0.553 |
| TC (mmol/L) | 3.96±0.96 | 4.12±1.16 | 4.10±0.99 | 0.520 | 4.13±1.04 | 4.04±1.03 | 4.10±1.10 | 0.834 |
| LDL (mmol/L) | 4.06±14.21 | 2.40±0.92 | 6.10±32.05 | 0.162 | 2.69±1.07 | 4.36±22.79 | 3.66±17.57 | 0.918 |
| HDL (mmol/L) | 1.12±0.27 | 1.11±0.27 | 1.12±0.25 | 0.897 | 1.07±0.27 | 1.11±0.26 | 1.12±0.27 | 0.854 |
| UA (μmol/L) | 302.71±91.24 | 298.49±91.99 | 303.37±93.60 | 0.851 | 348.62±95.67 | 298.22±91.05 | 300.17±92.33 | 0.164 |
| Urea | 5.24±1.78 | 5.40±2.35 | 5.36±1.95 | 0.836 | 5.79±3.51 | 5.22±1.82 | 5.41±2.19 | 0.536 |
| RBC | 4.27±0.85 | 4.24±0.97 | 4.18±1.03 | 0.758 | 4.26±0.65 | 4.11±1.04 | 4.27±0.95 | 0.265 |
| WBC | 10.02±14.58 | 10.40±13.74 | 11.67±15.72 | 0.607 | 8.04±3.00 | 11.62 ±15.09 | 10.51±14.54 | 0.594 |
| Platelet (10^9^/L) | 183.41±66.73 | 189.06±76.20 | 173.52±74.53 | 0.110 | 183.31±60.19 | 174.26±78.16 | 186.44±73.24 | 0.264 |
| HGB | 130.53±26.32 | 130.26±29.08 | 128.54±30.23 | 0.809 | 137.00±19.46 | 126.30±30.74 | 130.80±28.50 | 0.199 |
| Characteristics | **rs5017238** | | | | **rs5301** | | | |
|  | **GG** | **GA** | **AA** | *p* value | **TT** | **TC** | **CC** | *p* value |
| TG (mmol/L) | 1.65±1.07 | 1.49±0.79 | 1.56±0.66 | 0.277 | 1.49±0.58 | 1.55±0.88 | 1.65±1.05 | 0.574 |
| TC (mmol/L) | 4.10±1.10 | 4.01±1.02 | 3.97±0.95 | 0.657 | 4.05±1.03 | 4.04±1.03 | 4.10±1.10 | 0.885 |
| LDL (mmol/L) | 3.66±17.57 | 4.54±23.90 | 2.42±0.95 | 0.862 | 2.59±1.09 | 4.29±22.37 | 3.69±17.75 | 0.927 |
| HDL (mmol/L) | 1.12±0.27 | 1.11±0.25 | 1.10±0.33 | 0.917 | 1.08±0.26 | 1.11±0.26 | 1.12±0.27 | 0.890 |
| UA (μmol/L) | 299.84±92.14 | 297.57±92.84 | 333.89±94.47 | 0.270 | 344.43±93.24 | 297.74±89.98 | 300.32±93.01 | 0.193 |
| Urea | 5.40±2.19 | 5.28±1.85 | 5.49±2.98 | 0.835 | 5.73±3.39 | 5.24±1.81 | 5.40±2.21 | 0.614 |
| RBC | 4.27±0.95 | 4.13±1.05 | 4.11±0.88 | 0.346 | 4.28±0.63 | 4.10±1.05 | 4.27±0.94 | 0.189 |
| WBC | 10.52±14.54 | 11.52±14.73 | 11.26±14.83 | 0.797 | 8.24±2.98 | 11.77±15.24 | 10.43±14.50 | 0.525 |
| Platelet (10^9^/L) | 186.68±73.30 | 173.38±77.47 | 171.15±69.77 | 0.176 | 192.79±67.84 | 172.73±77.62 | 186.77±73.13 | 0.145 |
| HGB | 130.79±28.50 | 127.08±30.88 | 129.95±27.92 | 0.470 | 137.14±18.70 | 126.08±31.08 | 130.82±28.29 | 0.158 |

Abbreviations: TG, triglyceride; TC, total cholesterol; LDL, low-density lipoprotein; HDL, high-density lipoprotein; UA, uric acid; RBC, red blood cells; WBC, white blood cells; HGB, hemoglobin.

Additional File 1: Table S3 False positive report probability of the association *CYP11B1* polymorphisms and CHD susceptibility in subgroups

| Model and variables | Genotype | OR (95% CI) | *p^a^* | Statistical power | Prior probability | | | | |
| --- | --- | --- | --- | --- | --- | --- | --- | --- | --- |
|  |  |  |  |  | 0.25 | 0.1 | 0.01 | 0.001 | 0.0001 |
| **Female** |  |  |  |  |  |  |  |  |  |
| rs5283 A > G |  |  |  |  |  |  |  |  |  |
| Allele | A vs G | 1.37(1.01-1.85) | 0.040 | 0.993 | 0.108^b^ | 0.266 | 0.799 | 0.976 | 0.998 |
| Heterozygote | AG vs GG | 1.98(1.32-2.97) | 0.001 | 0.519 | 0.006 ^b^ | 0.016 | 0.155 | 0.649 | 0.949 |
| Dominant | AA+AG vs GG | 1.80(1.23-2.64) | 0.003 | 0.705 | 0.011 ^b^ | 0.032 | 0.270 | 0.788 | 0.974 |
| Additive | / | 1.40(1.03-1.91) | 0.034 | 0.988 | 0.093 ^b^ | 0.235 | 0.772 | 0.972 | 0.997 |
| rs6410 C > T |  |  |  |  |  |  |  |  |  |
| Allele | T vs C | 0.68(0.51-0.92) | 0.011 | 0.977 | 0.037 ^b^ | 0.103 | 0.557 | 0.927 | 0.992 |
| Homozygote | TT vs CC | 0.67(0.45-0.99) | 0.044 | 0.929 | 0.125 ^b^ | 0.301 | 0.825 | 0.979 | 0.998 |
| Dominant | TT+TC vs CC | 0.63(0.43-0.92) | 0.017 | 0.884 | 0.054 ^b^ | 0.146 | 0.653 | 0.950 | 0.995 |
| Additive | / | 0.66(0.49-0.90) | 0.009 | 0.960 | 0.026 ^b^ | 0.075 | 0.471 | 0.900 | 0.989 |
| **≤ 60 years** |  |  |  |  |  |  |  |  |  |
| rs6410 C > T |  |  |  |  |  |  |  |  |  |
| Allele | T vs C | 0.72(0.53-0.96) | 0.026 | 0.994 | 0.071 ^b^ | 0.186 | 0.715 | 0.962 | 0.996 |
| Heterozygote | TC vs CC | 0.67(0.45-0.99) | 0.042 | 0.929 | 0.125 ^b^ | 0.301 | 0.825 | 0.979 | 0.998 |
| Dominant | TT+TC vs CC | 0.65(0.45-0.94) | 0.023 | 0.918 | 0.067 ^b^ | 0.178 | 0.704 | 0.960 | 0.996 |
| Additive | / | 0.70(0.52-0.95) | 0.022 | 0.985 | 0.063 ^b^ | 0.168 | 0.689 | 0.957 | 0.996 |
| rs4534 C > T |  |  |  |  |  |  |  |  |  |
| Heterozygote | TC vs CC | 1.80(1.19-2.74) | 0.006 | 0.688 | 0.026 ^b^ | 0.074 | 0.468 | 0.899 | 0.989 |
| Dominant | TT+TC vs CC | 1.68(1.13-2.50) | 0.011 | 0.805 | 0.038 ^b^ | 0.105 | 0.564 | 0.929 | 0.992 |
| **Diabetes** |  |  |  |  |  |  |  |  |  |
| rs5283 A > G |  |  |  |  |  |  |  |  |  |
| Allele | A vs G | 1.45(1.09-1.93) | 0.012 | 0.986 | 0.032 ^b^ | 0.090 | 0.522 | 0.917 | 0.991 |
| Heterozygote | AG vs GG | 1.79(1.19-2.70) | 0.006 | 0.702 | 0.023 ^b^ | 0.066 | 0.437 | 0.887 | 0.987 |
| Dominant | AA+AG vs GG | 1.78(1.20-2.64) | 0.004 | 0.719 | 0.017 ^b^ | 0.049 | 0.363 | 0.852 | 0.983 |
| Additive | / | 1.47(1.09-1.98) | 0.011 | 0.979 | 0.033 ^b^ | 0.094 | 0.532 | 0.920 | 0.991 |
| rs4534 C > T |  |  |  |  |  |  |  |  |  |
| Allele | T vs C | 0.74(0.56-0.98) | 0.032 | 0.997 | 0.097 ^b^ | 0.243 | 0.780 | 0.973 | 0.997 |
| Homozygote | TT vs CC | 0.53(0.28-0.99) | 0.048 | 0.573 | 0.196 ^b^ | 0.422 | 0.889 | 0.988 | 0.999 |
| Additive | / | 0.73(0.54-0.98) | 0.036 | 0.994 | 0.099 ^b^ | 0.247 | 0.783 | 0.973 | 0.997 |
| **Hypertension** |  |  |  |  |  |  |  |  |  |
| rs4534 C > T |  |  |  |  |  |  |  |  |  |
| Homozygote | TT vs CC | 1.97(1.08-3.59) | 0.026 | 0.520 | 0.134 ^b^ | 0.317 | 0.836 | 0.981 | 0.998 |
| Recessive | TT vs TC+CC | 1.84(1.07-3.16) | 0.028 | 0.619 | 0.116 ^b^ | 0.283 | 0.813 | 0.978 | 0.998 |
| Additive | / | 1.33(1.01-1.75) | 0.044 | 0.998 | 0.111 ^b^ | 0.273 | 0.805 | 0.977 | 0.998 |

Abbreviations: CHD, coronary heart disease; OR: odds ratio; CI, confidence interval.

*p^a^ <0.05* indicates statistical significance.

^b^ The level of false positive report probability threshold was set at 0.2 and noteworthy findings are presented.
